# Increased Cytotoxic Efficacy of Protocatechuic Acid in A549 Human Lung Cancer Delivered via Hydrophobically Modified-Chitosan Nanoparticles As an Anticancer Modality

**DOI:** 10.3390/polym12091951

**Published:** 2020-08-28

**Authors:** Cha Yee Kuen, Tieo Galen, Sharida Fakurazi, Siti Sarah Othman, Mas Jaffri Masarudin

**Affiliations:** 1 Department of Cell and Molecular Biology, Faculty of Biotechnology and Biomolecular Sciences, Universiti Putra Malaysia, Selangor 43400, Malaysia; yeekuen910416cha@gmail.com (C.Y.K.); galenofficial94@gmail.com (T.G.); sarahothman@upm.edu.my (S.S.O.); 2 Department of Human Anatomy, Faculty of Medicine and Health Sciences, Universiti Putra Malaysia, Selangor 43400, Malaysia; sharida@upm.edu.my; 3 UPM-MAKNA Cancer Research Laboratory, Institute of Biosciences, Universiti Putra Malaysia, Selangor 43400, Malaysia

**Keywords:** hydrophobically modified-chitosan nanoparticle, protocatechuic acid, nanobiotechnology

## Abstract

The growing incidence of global lung cancer cases against successful treatment modalities has increased the demand for the development of innovative strategies to complement conventional chemotherapy, radiation, and surgery. The substitution of chemotherapeutics by naturally occurring phenolic compounds has been touted as a promising research endeavor, as they sideline the side effects of current chemotherapy drugs. However, the therapeutic efficacy of these compounds is conventionally lower than that of chemotherapeutic agents due to their lower solubility and consequently poor intracellular uptake. Therefore, we report herein a hydrophobically modified chitosan nanoparticle (pCNP) system for the encapsulation of protocatechuic acid (PCA), a naturally occurring but poorly soluble phenolic compound, for increased efficacy and improved intracellular uptake in A549 lung cancer cells. The pCNP system was modified by the inclusion of a palmitoyl group and physico-chemically characterized to assess its particle size, Polydispersity Index (PDI) value, amine group quantification, functional group profiling, and morphological properties. The inclusion of hydrophobic palmitoyl in pCNP-PCA was found to increase the encapsulation of PCA by 54.5% compared to unmodified CNP-PCA samples whilst it only conferred a 23.4% larger particle size. The single-spherical like particles with uniformed dispersity pCNP-PCA exhibited IR bands, suggesting the successful incorporation of PCA within its core, and a hydrophobic layer was elucidated via electron micrographs. The cytotoxic efficacy was then assessed by using an MTT cytotoxicity assay towards A549 human lung cancer cell line and was compared with traditional chitosan nanoparticle system. Fascinatingly, a controlled release delivery and enhanced therapeutic efficacy were observed in pCNP-PCA compared to CNP, which is ascribed to lower IC_50_ values in the 72-h treatment in the pCNP system. Using the hydrophobic system, efficacy of PCA was significantly increased in 24-, 48-, and 72-h treatments compared to a single administration of the compound, and via the unmodified CNP system. Findings arising from this study exhibit the potential of using such modified nanoparticulate systems in increasing the efficacy of natural phenolic compounds by augmenting their delivery potential for better anti-cancer responses.

## 1. Introduction

Despite improvements in the medical field nowadays, cancer remains one of the most studied diseases due to its complexity and continually increasing incidence rate throughout the decades. Global cancer statistics show lung cancer is the top cause for cancer-related deaths worldwide, with non-small cell lung cancer (NSCLC) accounting for approximately 84% of this statistic [1]. Current treatment for NSCLC includes surgery, chemotherapy, targeted therapies, immunotherapy, radiation therapy, and radiofrequency ablation therapy depending on the stage of cancer and other factors. However, even in curable NSCLC cases, death in patients can occur due to the onset of extensive distant metastases after initial treatment exercises [2]. The most common treatment regime for NSCLC includes chemotherapy utilizing therapeutic agents such as Carboplatin, Cisplatin, Paclitaxel (Taxol), and Gemcitabine (Gemzar). According to the National Cancer Institute, the use of such drugs in anticancer therapy will frequently confer unwanted side effects, such as hair loss, fatigue, anemia, appetite changes and nausea, and vomiting, which further hinder the recovery of patients. These Food and Drug administration (FDA)-approved drugs can give rise to these side effects frequently due to the high dose administration and non-specific destruction of these chemotherapy agents, where the non-specific cytotoxicity often resulted in low tumor specificity and high toxicity, leading to various concomitant side effects [3]. Therefore, alternatives for these chemotherapy agents are needed to avoid unpleasant side effects for the patients.

In lieu of this, extensive research has been conducted in search of derivative- and natural-based compounds that can be used as alternatives for traditional chemotherapy drugs [4,5]. It is more beneficial to use natural anti-cancer compounds as compared to chemotherapy agents as they can aid in reducing these concomitant side effects and reduce discomfort in patients [6]. This was suggested by Demain and Vaishnav as natural compounds, including curcumin, isoflavone genistine, and resveratrol, induced apoptosis death of cancer cells without any adverse effect on normal cells [7]. Previous studies have shown that phenolic compounds, such as green tea extract [8,9], curcumin [10,11], and caffeic acid [12,13], are among natural compounds that possess potent anticancer effects against lung carcinomas. Among these alternates, protocatechuic acid (PCA) is a natural phenolic compound broadly distributed in most edible plants utilized for folk medicine [14,15]. PCA has been reported to be anti-bacterial [16], anti-oxidative [17], anti-cancer [18], anti-diabetic [19], anti-ageing [20], and anti-inflammatory [21]. Yin et al. have suggested that PCA has revealed an anticancer effect towards human cancer cells, including lung, breast, liver, and prostate cancer cells through apoptosis or the suppression of invasion and metastasis of the cancer cells [22]. Moreover, evidence from Hu et al. shown that PCA at 25 µM concentration has significantly inhibited vascular endothelial growth factor (VEGF)-induced cell proliferation of human umbilical vein endothelial cell (HUVECs) by 22.68 ± 5.6% assessed by an MTT assay which further suggested PCA as a candidate treatment for cancer tumors [23]. Apart from that, a previous study of Tsao et al. has reported that PCA treatments at 2–8 µM were able to inhibit the cell growth of lung cancer A549, H3255, and Calu-6 cells in a dose-dependent manner through modulation of FAK, MAPK, and NF-kB pathways, and downregulation of the protein production of growth factors proposed PCA as a good candidate for lung cancer therapeutics [24]. However, PCA, which is also commonly known as 3,4-dihydroxybenzoic acid, possesses sparingly a solubility of 1:50 ratio in water. This hampers its use in the medical field, including in cancer treatment, since the solubility may directly affect its absorption and bioavailability [25]. Consequently, the efficiency of PCA as a therapeutic agent can be potentiated by improving its cellular delivery and uptake. By increasing its accumulation through higher cellular uptake, the efficacy of PCA can be potentially increased while minimizing adverse responses associated with the many side-effects of more potent chemotherapy drugs.

One possible strategy to increase its efficiency is by optimizing its uptake and delivery into cancer cells. Oral delivery is the most popular and economical administration route for therapeutics but requires overcoming biological barriers such as absorption, solubility, and dissolution, pre-systemic metabolism, and excretion [26]. Parenteral delivery is the most simple and convenient drug delivery system but involves the application of specialized tools and techniques to arrange and administer parenteral formulations [26]. Subsequently, poor cellular uptake of the phenolic compounds has also led to a high dose of therapeutic administration, thus conforming to a restricted therapeutic value due to issues of dose-dependent morbidities [27]. To overcome these problems, research has focused on attempts in assisting or enhancing current drug delivery systems. This has included the adaptation of nanoparticulate delivery systems to complement established oral and parenteral delivery systems [28]. The utilization of nanoparticles for the encapsulation of cargos such as various therapeutic drugs or compounds and genetic materials have been reported by innumerable researchers over the years. Various nanoparticle systems have been formulated by the researchers, including metallic, liposome, carbon nanotube, solid lipid, and polymeric nanoparticle systems. These nanoparticle systems vary in their physical and surface properties due to the features of their respective building materials [29]. Nonetheless, they have shared some crucial common characteristics where they have sizes of less than 100 nm at least in one of the three dimensions and are capable of encapsulating cargos [30]. In recent decades, nanotechnology has emerged as one of the promising tools in various sectors, including cosmetics, electronics, food, and agriculture, as well as biomedical and pharmaceutical fields [31]. The application of nanobiotechnology in cancer therapy has been incorporated into several treatments such as hyperthermia, gene therapy, and targeted cancer therapy. A previous study of Giustini et al. showed that utilization of magnetic nanoparticles in hyperthermia cancer treatment was advantageous in achieving an enhanced permeability and retention (EPR) effect and achieved targeted delivery [32]. Additionally, Wu et al. described a SP94 peptide-conjugated PEGylated liposomal doxorubicin towards human hepatocellular carcinoma cell lines and revealed a significant drug accumulation increment in tumors in comparison to non-targeted PEGylated liposomal doxorubicin by about 8.8-fold greater cellular uptake in SK-HEP-1 cells, and revealed greater therapeutic effects in both in vitro and in vivo studies [33].

Several nanoparticle formulation systems have been shown to aid in delivery purposes, including metal nanoparticles, carbon-based nanoparticles, polymeric nanoparticles, as well as lipid-based nanoparticles [34,35]. However, their eventual adaptation for anticancer modalities are often constricted to issues of inherent toxicity and robust synthesis regimes. For example, titanium dioxide (TiO2) nanoparticles have been demonstrated to profusely accumulate in the mouse hippocampus post-administration to affect hippocampal apoptosis and damage in spatial recognition memory [36]. Carbon-based nanoparticles potentially induce oxidative stress, as shown by Wang et al. in that single-walled carbon nanotubes exerted significant cytotoxicity towards rat PC12 cells in a wide dose range of 5–600 µg/mL for 24 and 48 h [37]. Previous studies have described that the presence of transition metals in carbon nanotubes induces the formation of molecular oxygen-dependent superoxide anion radicals, hydroxyl radicals, and hydrogen peroxide, which have high redox potentials and reactivities [38,39]. On the other hand, biodegradable polymeric nanoparticles serve as a good candidate vector to develop anticancer modalities with additional sustained release properties while being biocompatible with cells and tissues [40]. Chitosan nanoparticles (CNP) constitute a polymeric nanoparticle system that has been commonly reported for drug delivery applications. Both the amine (–NH_2_) and hydroxyl (–OH) groups of chitosan are active spots for modification to initiate different modification requirements [41]. CNP has been modified using emulsification solvent diffusion methods to increase the entrapment of hydrophobic drugs [41,42]. Glycol-chitosan has been hydrophobically modified through chemical conjugation using hydrophobic 5β-cholanic acid moieties and the hydrophilic glycol chitosan backbone to encapsulate water-insoluble camptothecin (CPT) into the hydrophobically modified glycol chitosan nanoparticles with high loading efficiency with a sustained release property [43]. These previous modifications have therefore led to the modification of chitosan in this current study using palmitic acid to synthesize hydrophobically modified-chitosan nanoparticles (pCNP) to increase the encapsulation of PCA in pCNP through hydrophobic–hydrophobic interactions, and in turn to enhance the therapeutic efficacy of PCA in A549 lung cancer cell treatment.

**Figure 1:**
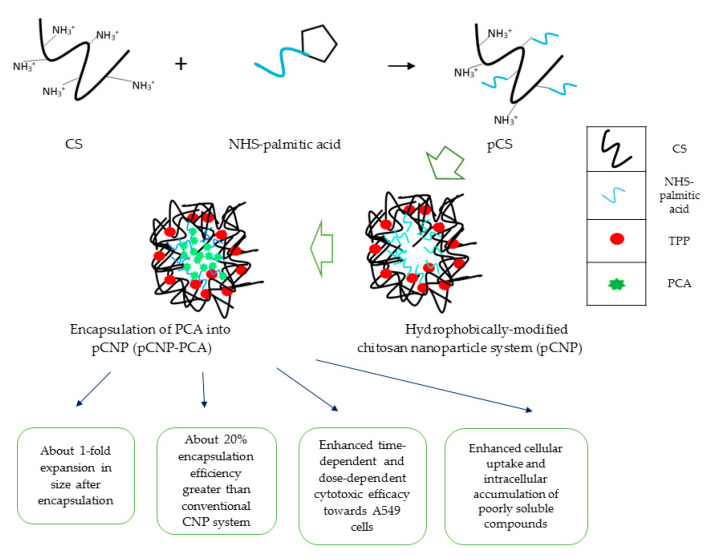
Overview of the study.

This current study describes the enhanced therapeutic response and controlled release property of the phenolic acid PCA in the A549 human lung cancer cell line mediated through its encapsulation in hydrophobically modified chitosan nanoparticles (pCNP), as compared with conventional CNP systems. The overview of this study is shown in Figure 1, where a hydrophobic anchor based on palmitoyl was conjugated to chitosan polymer via NHS-ester bridges, and the resulting nanoparticles were characterized via various physicochemical analyses. This novel pCNP system is suggested as a safe and effective alternative nanocarrier system for the enhanced therapeutic delivery of PCA, which could be a potential nanocarrier system for other poorly soluble therapeutics. The findings from this research are expected to aid in the enhancement of PCA for anti-cancer applications.

## 2. Materials and Methods

Chitosan (CS, low molecular weight), sodium tripolyphosphate (TPP), palmitic acid N-hydroxy-succinimide ester (NHS-palmitate), protocatechuic acid (PCA), and dimethyl sulfoxide (DMSO) were acquired in powder form from Sigma-Aldrich (St. Louis, MO, USA). Roswell Park Memorial Institute-1640 medium (RPMI-1640), fetal bovine serum (FBS), 0.25% trypsin-EDTA (1×), and Antibiotic-Antimycotic (100×) were purchased from Gibco Life Technologies (Grand island, NY, USA). Glacial acetic acid, sodium hydroxide, and hydrochloric acid (analytical grade) were obtained from Friendemann Schmidt Chemicals (Parkwood, Western Australia). All reagents, unless otherwise stated, were used without further purification.

### 2.1. Formation of Chitosan Nanoparticles (CNP)

CNPs were prepared by ionic gelation route as previously described by Masarudin et al. [44]. Chitosan (CS) and Tripolyphosphate (TPP) were prepared to a concentration of 1.0 mg/mL in 50 mL centrifuge tubes and further diluted to 0.5 and 0.7 mg/mL respectively and adjusted to pH 5 and pH 2 using 1 M NaOH and 1 M HCl. Subsequently, nanoparticles were formed by adding increasing volumes of TPP solution (0 to 300 μL) to 600 μL of CS solution. The CNPs were purified by centrifugation at 13,000 rpm for 20 min. After that, 40% of the total CNPs supernatant volume were mixed with 60% of deionized water (dH_2_O) corresponding to the 40% supernatant volume and used for further analyses.

### 2.2. Hydrophobic Modification of Chitosan Nanoparticles (pCNP)

Hydrophobic modification was performed by the spontaneous conjugation of palmitoyl groups to the CS backbone prior to nanoparticle formation. Initially, 1.0 mg/mL CS solution was adjusted to pH 6. Separately, NHS-palmitate was prepared in absolute ethanol to a concentration of 0.9 mg/mL. The NHS-palmitate solution was subsequently added to the CS solution by dropwise additions at a 2:1 volume ratio and the conjugation reaction was left to occur a further 20 h at 50 °C. Following incubation, hydrophobically modified chitosan (pCS) was precipitated from the mixture by adjusting the pH to 9. It was then centrifuged at 4500 rpm for 45 min to separate the precipitate from the solution. The precipitate was washed once with an acetone: ethanol (50:50) solution, and successively thrice with dH_2_O before being dried in oven at 50 °C. The pCNP was prepared therewith using similar methods as described for CNP.

### 2.3. Synthesis of Protocatechuic Acid-Encapsulated Nanoparticles (CNP-PCA and pCNP-PCA)

Approximately 1.5 mg of PCA was dissolved in 2 mL dH_2_O and allowed to stir at 60 °C for approximately 10 min using a magnetic stirrer to prepare a 5 mM master stock. To form PCA-encapsulated nanoparticles, 200 µL of PCA was mixed with 600 µL of CS/pCS followed by 200 µL of TPP. The resulting CNP-PCA and pCNP-PCA were then directly used for consequent physicochemical analyses.

### 2.4. Physicochemical Characterization of Nanoparticles

Particle size by intensity and polydispersity index (PDI) of nanoparticle samples (CNP, pCNP, CNP-PCA and pCNP-PCA) were determined using dynamic light scattering on a Malvern Zetasizer Nano S Instrument (Malvern Instruments, Malvern, UK). Approximately 1000 µL of sample was aliquoted into a disposable cuvette and analyzed in triplicate to ensure the stability of the samples. All the data were recorded as mean ± standard error of mean (SEM). Surface morphology of nanoparticles were assessed using field emission-scanning electron microscopy (FESEM). The samples were first diluted prior to analysis by mixing 100 μL of the samples with 500 μL of dH_2_O. Then, a single drop of each diluted samples was coated onto an aluminum stub and left to dry in an oven for at least 3 days. Next, vacuum gold-coating was performed for the sample-loaded stubs before observation under a FEI NOVA nanoSEM 230 electron microscope. Internal surface of the nanoparticle samples was examined using transmission electron microscopy (TEM). For TEM analysis, the diluted samples were drop-coated directly onto copper grids and dried under a hot light bulb before observation under a TECNAI G2 F20, FEI TEM. Determination of characteristic functional groups in samples were performed using a Spectrum 100 Perkin-Elmer FTIR instrument. Prior to analysis, all samples were freeze dried in a Coolsafe 95-15 PRO freeze drier (SCANVAC, Lynge, Denmark) for 48 h. The samples were analysed using attenuated total reflectance (ATR) at an infrared frequency range of 200–4000 cm^−1^.

### 2.5. Determination of Free Amine Groups Using Trinitrobenzene Sulfonic Acid Assay (TNBS)

Free amine groups in chitosan was determined to ascertain conjugation reactions with NHS-palmitate, and successful formation of nanoparticle samples. Precedingly, solutions of 0.05% (*v*/*v*) TNBS reagent, 1.0 M HCl, 10% (*w*/*v*) SDS and 0.1 M (*w*/*v*) NaHCO_3_ were separately prepared in 15 mL centrifuge tubes. Then, a chitosan standard solution was prepared by serially diluting 50 μL CS solution (0.5 mg/mL) using 0.1 M NaHCO_3_. About 50μL of 0.05% (*v*/*v*) TNBS solution was then added to each CS/pCS sample in 0.5 mL centrifuge tubes. For sample solutions, 100μL of nanoparticle samples at different TPP volume addition was mixed with 100 µL 0.05% (*v*/*v*) TNBS solution in centrifuge tubes. All tubes were then incubated in a water bath for 3 h at 37 °C. Subsequently, 100 μL of the standard/sample solutions were transferred into a 96-well plate and mixed with 100 μL of 10% (*w*/*v*) SDS and 75 μL of 1 M HCl respectively. The absorbance was then read at A_335nm_ and the utilized amine percentage was calculated using the following equation:

$$100-(\mathrm{Free}\;\mathrm{amine}\;\mathrm{percentage}\;(\%)=\frac{A_{335}\;\mathrm{of}\;\mathrm{CNP}/\mathrm{pCNP}}{\begin{array}{c} A_{335}\;\mathrm{of}\;\mathrm{CS}/\mathrm{pCS}\\ \left(\mathrm{at}\;\mathrm{same}\;\mathrm{concentration}\;\mathrm{used}\right) \end{array}}\;\times \;100\%)$$

#### Determination of PCA Encapsulation Efficiency (%EE) in Nanoparticle Samples:

The encapsulation efficiency (% EE) was analyzed by comparing the difference in absorbance at A_296nm_ between free PCA and the supernatant of encapsulated PCA. The nanoparticles samples were prepared as previously described. The samples were centrifuged at 18,000 rpm for 30 min. The supernatant of each sample was then collected, and the absorbance was read at A_296nm_ using an Implen NP80 U*V*/*V*IS spectrophotometer. The % EE was calculated using the following equation:

$$\%\;EE=\frac{A_{296}{\;\mathrm{of}\;\mathrm{free}\;\mathrm{PCA}\;-\;A}_{296}\;\mathrm{of}\;\mathrm{PCA}\;\mathrm{in}\;\mathrm{supernatant}}{A_{296}\;\mathrm{of}\;\mathrm{free}\;\mathrm{PCA}}\;\times \;100\%$$

### 2.6. Assessment of In Vitro Vellular Efficacy of Nanoparticle Mediated PCA Uptake in A549 Lung Cancer Cell Line

The A549 lung cancer cell line was established and maintained by aseptic cell culture regimes in a T-25 flask with growth media consisting of 90% of 1X RPMI medium 1640 and 10% (FBS). The flask was maintained in incubator at 37 °C, supplied with 5% CO_2_ and 90% humidity. About 100 μL of cells were seeded onto a 96-wells plate. The cells in 96-wells plate were treated with 100 μL of CNP, pCNP, PCA, CNP-PCA, and pCNP-PCA at different concentrations. At the end of each time point, the old media in each well were decanted and replaced with 170 μL fresh media solution and 30 µL of 5 mg/mL MTT solution. After 4 h incubation at 37 °C, all the solution in the wells was removed and replace with 100 μL DMSO. The absorbance was then read at *A*_570nm_ on a Bio-Rad iMark™ Microplate Absorbance Reader. Cell viability was then determined using the following equation:

$$\%\;\mathrm{Viability}=\frac{A_{570}\;\mathrm{of}\;\mathrm{treated}\;\mathrm{cells}}{\begin{array}{c} A_{570}\;\mathrm{of}\;\mathrm{untreated}\;\mathrm{cells}\\ \left(\mathrm{at}\;\mathrm{same}\;\mathrm{concentration}\;\mathrm{used}\right) \end{array}}\;\times \;100\%$$

## 3. Results and Discussions

### 3.1. The Colume of Cross-Linker Governing the Size and PDI of Nanoparticles

**Figure 2:**
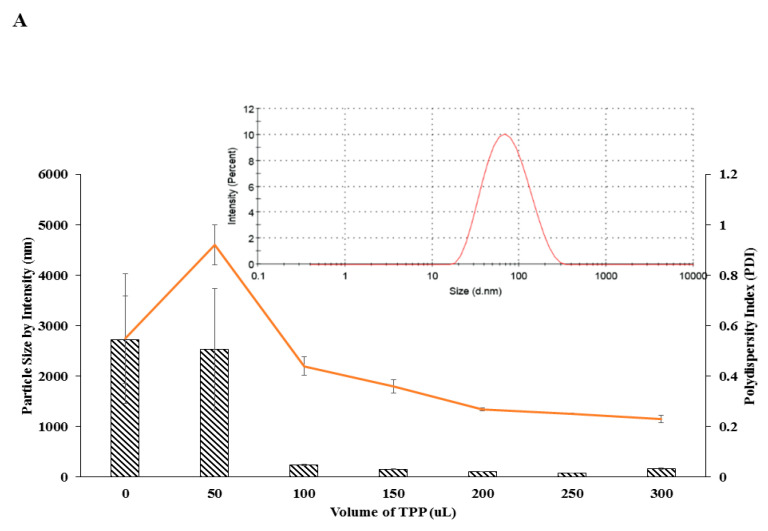

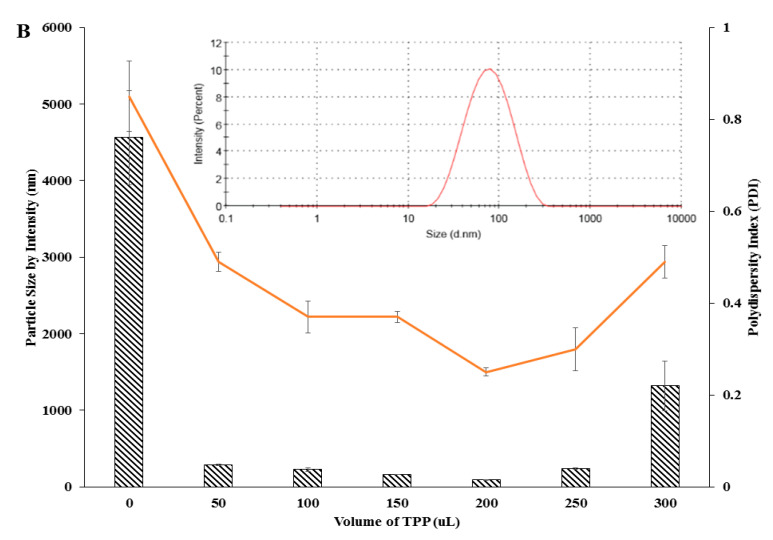
The PSD and PDI value of (**A**) CNP and (**B**) pCNP at different TPP volumes. Represented by bar (particle size) and line (PDI value) graphs. The smallest size of 82.24 ± 2.67 nm was obtained at 250 µL of TPP for CNP with PDI of 0.25 while 90.23 ± 2.67 nm at 200 µL for pCNP with PDI of 0.25. The DLS graph of CNP and pCNP synthesized by using 250 µL and 200 µL was shown inset in both Figure 2A,B, respectively. Error bars represent the SEM averaged from three independent experiment replicates. One-way ANOVA was performed with *p* > 0.05 for both particle size and PDI indicating no significant difference between the three experimental replicates.

The nanoparticles were spontaneously formed through the cross-linking of amine groups of chitosan polymer and phosphate groups of the cross-linker, TPP [45]. As shown in Figure 2A,B, nanoparticle size conferred a decreasing trend with increasing TPP volume until an optimum CS:TPP volume ratio was reached. Initially, when no TPP was added to the CS, the size of the polymer was 2720.33 ± 870.26 nm and slightly decreased to 2534.00 ± 1203.00 nm at 50 µL TPP volume addition, and significantly dropped to 241.57 ± 16.29 nm at 100 µL TPP volume addition. It was then gradually decreased to the smallest size upon 250 µL TPP volume addition. A similar trend was revealed by pCNP where the size of initial pCS at no TPP addition was 4560.33 ± 614.17 nm, dramatically dropped to 289.83 ± 8.92 nm at 50 µL TPP volume addition, and gradually decreased until it reached its smallest size of 90.23 ± 2.67 nm at 200 µL TPP addition. It showed that the minimum volumes of TPP required for a nano-sized particle to form were 100 µL and 50 µL for CNP and pCNP respectively. The initial size of CNP at 100 µL TPP volume addition was 241.57 ± 16.29 nm while pCNP at 50 µL TPP volume addition was 289.83 ± 8.92 nm. The smallest nanoparticle size of CNP obtained from this study was 82.24 ± 2.67 nm which by using 250 uL TPP while PCNP was 90.23 ± 2.67 nm by using 200 uL TPP. This result was congruent with the findings of Kavi Rajan et al. where the optimum chitosan to TPP ratio of about 3:1 [46]. Thereafter, particle size increased exponentially, indicating the formation of aggregates and nanoparticle clusters after this threshold resulting in the existence of excess TPP in the aqueous system, which may promote further interaction between the CNPs/pCNPs, thus initiating agglomerated nanoparticles with larger sizes [47,48]. Similarly, PDI values followed a similar decreasing trend with increased TPP volume. As shown in Figure 2A, the initial PDI value of CNP was 0.55 ± 0.25 and subsequently increased to 0.92 ± 0.08, and then decreased to 0.44 ± 0.03, 0.36 ± 0.03, 0.27 ± 0.01 until it reached the lowest point of 0.25 ± 0.01 at 250 µL of TPP addition. Next, the initial PDI value of pCNP was 0.85 ± 0.08 and decreased gradually to 0.49 ± 0.02, 0.37 ± 0.04, 0.37 ± 0.01 until it reached its lowest point of 0.25 ± 0.01 at 200 µL TPP volume addition. The previous study of Masarudin et al. revealed that the addition of 20 µL of TPP into 600 µL of CS has initiated the formation of nano-scale CNP and subsequently reached the smallest size with 200 µL of TPP, which is comparable to our current study [1]. Besides that, the PDI of 0.2–0.3 indicated the uniformity of the nanoparticles formed by the 3:1 CS/pCS to TPP volume ratio, which is supported by the findings of Koukarous [49]. This decreasing trend of size and PDI across both CNP and pCNP with increased TPP volumes addition was suggested due to the increased availability of the cross-linker to interact with the free amino groups existed in the fixed volume of chitosan polymer. Interestingly, despite the similar lowest PDI obtained by both CNP and pCNP, it was observed that the smallest size of pCNP was slightly larger than that of CNP by about 9.72%. This finding coincided with the previous study of Farhangi et al., where the conjugation of fatty acid chains into chitosan will result in an increased in size of the nanosystem [50]. This finding is comparable with our study, in which the conjugation of palmitic acid in the CS will correspondingly slightly increase in the size of nanoparticles due to the conjugation of extra component in CS polymer. Nonetheless, this insignificant particle size increment is expected and acceptable since the size of pCNP was still below 100 nm.

### 3.2. The Formation of Nanoparticles Utilized Free Amine Group of CS/pCS Polymer

**Figure 3:**
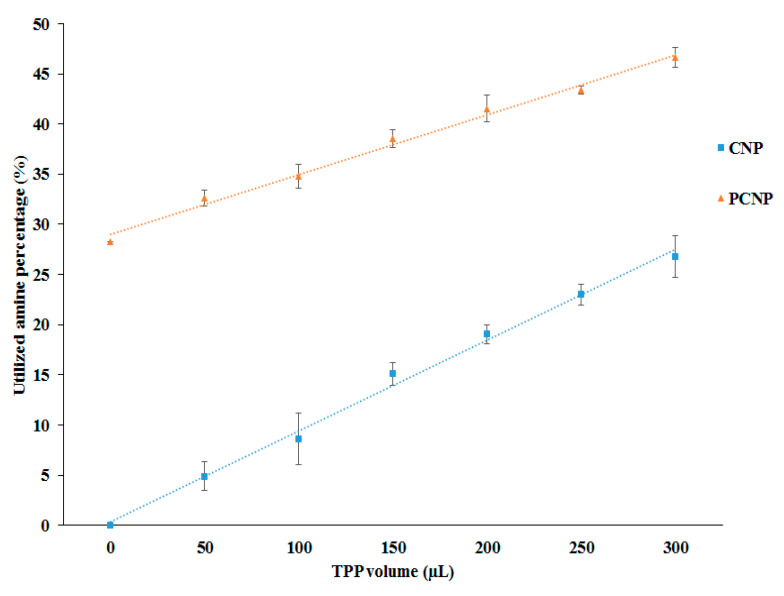
The utilization of amine percentage with different TPP volumes. The utilization of free amine percentage increased with increased TPP volume for CNP and pCNP. Data presented as mean ± SEM from three independent experiment replicates. Two tailed paired *t*-test was performed with *p*-value < 0.0001.

The TNBS assay is a well-known assay to quantify the free amine group as described earlier by Satake et al. [51]. As the formation of nanoparticles occurred through cross-linking between the cationic polymer and anionic cross-linker, amine group utilization was expected to show a decreasing value during conjugation and particle formation reactions. As shown in Figure 3, the utilization of amine groups increased following the increased TPP volumes used for both CNP and pCNP samples. In CNP samples, amine utilization of up to 26.75 ± 2.06% was achieved, while pCNP showed approximately 46.64 ± 0.94% amine utilization when a maximum of 300 µL TPP volume was used for cross-linking. Expectedly pCNP was shown to utilize a significantly higher percentage of amine groups compared with CNP. Considering that pCNP precedingly involved the conjugation of NHS-palmitoyl to CS prior to nanoparticle formation with TPP, an increase in its amine utilization suggested that the conjugation of palmitic acid was successfully obtained through the utilization of approximately 28.29% of amine groups. Data presented are similar to previous studies which postulate that the formation of CNP and pCNP will utilize the amine group of CS polymer and with greater utilization percentage in pCNP formation [52]. This result indicated a proportional increment in amine utilization with increased volume of the crosslinker regardless of their size and PDI value. It is because the formation of pCNP aggregates also happened through the cross-linking between the nanoparticles and excess TPP cross-linker which will further increased the amine utilization [53]. The pCNP has a greater utilization than CNP in all measured data, due to the utilization of amine groups through the conjugation of -NHS palmitic acid to the amine groups of chitosan. The study of Esquivel et al. reported a synthesis of thiol-modified chitosan with a utilization of 11% of amine groups in chitosan which is similar to our study that has utilized around 15% of free amine in chitosan following –NHS palmitic acid conjugation [54]. Besides that, the literature described by Mohammed et al. also proposed that chemical modifications of chitosan such as amphiphilic chitosan, carboxylated chitosan, and lactose-modified chitosan were achieved through the reaction between amine groups of chitosan and the modifying agents [41]. Thus, it can be deduced that the utilization of free amine percentage by pCNP will appear higher than CNP due to the utilization by the modification step. In addition, the excess percentage of amine utilization by pCNP than CNP was considered acceptable since there is still room for the cross-linking with TPP.

### 3.3. Formation of PCA-Encapsulated Nanoparticles

**Figure 4:**
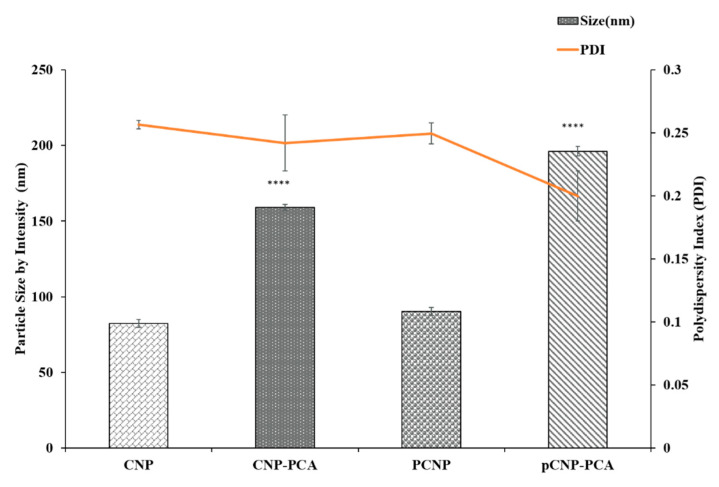
The expansion of nanoparticle size and PDI value after encapsulation. The results show that the size of empty nanoparticles expanded after encapsulation. Data are presented as mean ± SEM from three independent experiment replicates. One-way ANOVA was performed with **** *p* < 0.0001 indicating the significant difference in size between both CNP-PCA and pCNP-PCA with CNP and pCNP.

PCA was successfully encapsulated in both CNP and pCNP following spontaneous formation of nanoparticles after crosslinking with TPP. Encapsulation led to an increase in particle size to accommodate the phenolic compound within its internal structure. As shown in Figure 4, CNP-PCA particle size expanded 93.4% from 82.2 to 159.0 nm, whilst comparatively, a larger expansion was observed in its hydrophobically-modified counterpart. The particle size of pCNP-PCA increased from 90.2 to 196.3 nm, a 117.6% surge from empty pCNP. As this expansion correlated with previous study [46], the inclusion of a hydrophobic moiety within pCNP-PCA has affected its expansion compared to CNP-PCA at similar PCA concentrations used for encapsulation. This was ascribed to an increased amount of PCA encapsulated in pCNP-PCA, due in part towards a tighter hydrophobic-hydrophobic interaction forming between the palmitoyl groups in pCS with PCA prior to nanoparticle formation. Such interactions have also been similarly been reported by Wang et al., where they have conducted hydrophobic modification of chitosan using cholesterol conjugate through succinyl linkages and successfully increase the encapsulation efficiency of poorly water soluble epirubicin, an anthracycline topoisomerase inhibitor from 7.97% to 14.0% [55]. Since this palmitoyl anchor is absent in CNP, encapsulation reactions did not benefit from this extra interaction and were thus lower in terms of the amount of PCA within the nanoparticle core after formation. This correlated to a substantial difference in *% EE* values between CNP-PCA and pCNP-PCA as well, which further illustrates the enhanced compound loading properties following hydrophobic modifications of the nanoparticle.

The % EE of PCA in CNP-PCA and pCNP-PCA has shown in the Table 1. The encapsulation efficiency of PCA was approximately 35.2 ± 1.7% in CNP-PCA, while pCNP-PCA had a significantly higher % EE of 54.4 ± 3.9%. A higher encapsulation efficiency was attained in pCNP-PCA as compared with CNP-PCA, which was mostly due to the hydrophobic anchor acquired by the presence of palmitoyl that associated with the modified pCS polymer, and consequently enable a higher encapsulation of PCA upon nanoparticles formation. This observation also correlated to a higher degree of expansion in the hydrophobically-modified nanoparticles, as described previously. Several studies have also reported enhanced encapsulation properties in hydrophobically-modified chitosan nanoparticles using other hydrophobic moieties such as deoxycholic acid, stearyl, phthaloyl, and N-acetyl histidine [56,57,58,59]. Zhang et al. also demonstrated that the hydrophobically modified chitosan nanoparticle was able to encapsulate the Doxorubicin to act as a carrier system for antitumor agents [60]. Previous literature studies suggested that the modification of chitosan by long alkyl chains (C6-C12) will gradually promote a more efficient hydrophobic interactions and intra-aggregation corresponding to the length of alkyl chains as compared with short alkyl chains (C5) [61]. In correlation with this study, palmitic acid with long alkyl chain (C15) was suggested to incur efficient hydrophobic interactions with PCA. Furthermore, Ways et al. have also highlighted that various chemical modifications of chitosan including trimethyl chitosan, thiolated chitosan, acrylated chitosan and acetylated chitosan will mainly occasioned in an enhancement in the loading, bioavailability and a substantial improvement of the therapeutic efficacy of some candidate drugs compared to unmodified chitosan [62]. The higher *% EE* attained by pCNP-PCA compared to CNP-PCA indicated a greater amount of PCA being encapsulated in pCNP, which may indirectly increase the therapeutic efficacy of pCNP-PCA. This assumption was supported by the previous study of Ong et al. where a greater % EE will enhance the bioavailability and consequently improved the absorption of encapsulated compounds as compared to their lower *% EE* counterpart [63]. Although the maximum % EE of pCNP-PCA was not assessed in this current study, it was suggested that *% EE* of pCNP-PCA may be improved by other strategies including dual or multiple loading of cargos [64]. However, these approaches may include complex and tedious procedures which may change the native structure of the therapeutic which may in turn affect its therapeutic efficacy [65].

The PDI values of both CNP-PCA and pCNP-PCA were measured as of 0.20 ± 0.02 to 0.25 ± 0.01, as shown in Figure 4. This implied that the nanoparticle samples occurred at a high monodispersity and reproducibility [66]. Similarly, the hydrophobic modification in pCNP-PCA did not affects its dispersity. This suggested that the expansion of the size between both types of nanoparticles was almost similar. This postulation was parallel with the previous study of Maruyama et al. where the encapsulation of herbicides imazapic and imazapyr into CNP did not significantly alter the PDI of the system which indicating the homogeneity and stability of the nanoparticle system after encapsulation [67]. Additionally, the previous study of Othman et al. also signified that dual-loading of L-ascorbic acid and thymoquinone into CNP system has obtained similar PDI values of 0.19 ± 0.02 prior to, and 0.21 ± 0.01 after encapsulation has also suggested that encapsulation of therapeutics into CNP system not necessarily altered the PDI values [64]. In correlation with these findings, it was proposed that an equal or almost equal distribution of PCA occurred in both pCNP and CNP samples which resulted in an almost similar PDI being obtained in both systems.

### 3.4. Morphological Analysis of Nanoparticles

**Figure 5:**
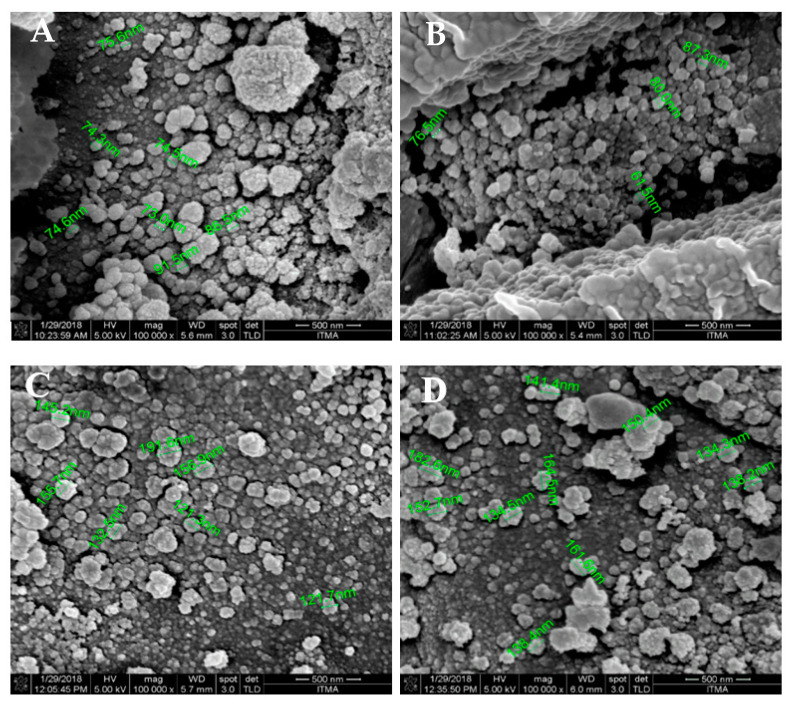
FESEM analysis of nanoparticle samples. (**A**) CNP, (**B**) pCNP, (**C**) CNP-PCA and (**D**) pCNP-PCA. The nanoparticle samples were revealed in single-spherical like particles with uniformed dispersity with different size range.

The morphological properties of CNP-PCA and pCNP-PCA was studied by using FESEM and TEM. Figure 5 showed the presence of single-spherical like particles with uniformed dispersity in both hydrophobically-modified and non-modified nanoparticles, with its approximate size correlating with DLS data. Particle size of CNP ranged from 73.0 to 91.5 nm for CNP (Figure 5A), while pCNP was in the range of 61.5 to 87.3 nm (Figure 5B). The morphology of pCNP was smooth and single-spherical like particle in shape and comparable with CNP, suggesting that the conjugation of palmitic acid in pCNP has no contributions to the surface morphology. This observation was expected since the palmitoyl group was likely to avoid from the surrounding water environment and resides in the interior of the pCNP [68]. Nevertheless, a population of pCNP with smaller sizes than CNP was observed in the figure. This qualitative morphology involved a randomly chosen site which was in contrast to the mean size of nanoparticles earlier, while the size reflected by the morphology was a relative approximation of nanoparticle size. The DLS measured the size average across all size populations while FESEM imaging observed at random spots which might be resulted in nanoparticles with sizes slightly deviated from the mean value obtained by DLS analysis [69]. Additionally, the difference in sizes may also due to the fundamental difference in the preparation of these two techniques, where the samples in DLS are hydrated, whereas in FESEM they are under vacuum, which will clearly have an important impact on the sizes measured. The morphology of CNP-PCA and pCNP-PCA was also observed as smooth and single-spherical like particles in shape in which similar to the blank nanoparticles. Meanwhile particle size upon PCA encapsulation showed an increased for both CNP-PCA and pCNP-PCA samples, which ranged from 121.3 to 191.6 nm for CNP-PCA (Figure 5C) and from 138.2 to 182.6 nm for pCNP-PCA (Figure 5D), which indicated interrelated measurements with data from DLS analysis. Moreover, a similar morphology was noticed in both CNP-PCA and pCNP-PCA which consisted a range of size populations. Although the *% EE* was higher in pCNP-PCA, the expansion of size between the CNP-PCA and the former is approximately not that significant. This observation suggested that the utilization of greater amine groups in pCS/pCNP due to conjugation of palmitic acid resulting in a lower net positive charge, together with the more specific hydrophobic-hydrophobic interactions between pCNP and PCA aid in developed pCNP-PCA of more compact nanoparticles and thus smaller in size, which explained the expansion of size of pCNP-PCA as comparable with CNP-PCA where an even higher % EE was attained [70].

**Figure 6:**
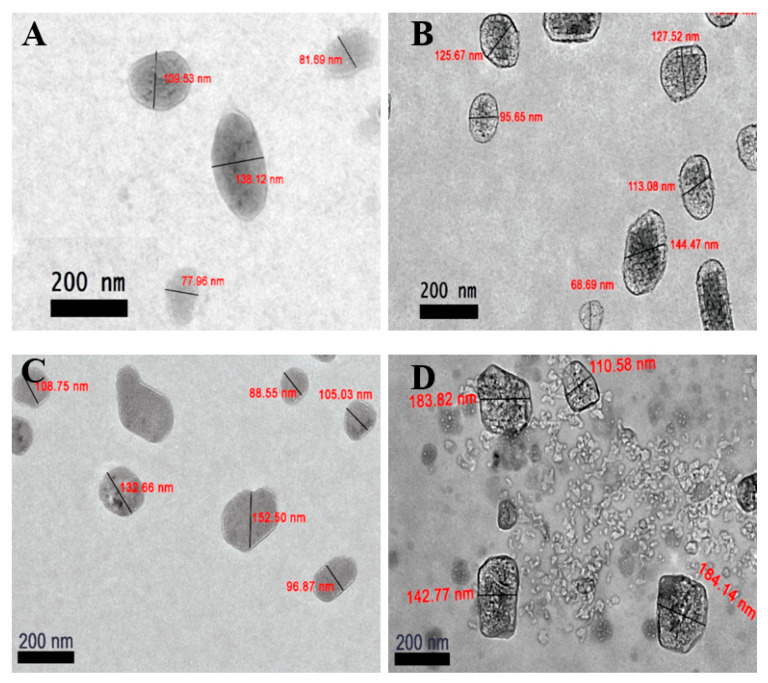
TEM analysis of nanoparticle samples. (**A**) CNP, (**B**) pCNP, (**C**) CNP-PCA and (**D**) pCNP-PCA. The nanoparticle samples were distributed with single-spherical like nanoparticles with uniformed dispersity with different size range.

Conversely, Figure 6 showed the phase morphology of the nanoparticle samples. As mentioned by Mayeen et al., the electrons in TEM can penetrate through the samples and measures the changes of the electron beam to assess the internal structure of the samples; while FESEM works by scanning through the surface of samples through a raster scan pattern to assess the surface morphology of samples [71]. This feature was supported by Barhoum and García-Betancourt who further proposed FESEM and TEM to use in the morphology characterization analysis of nanoparticles to provide a detailed characterization of nanostructures [72]. TEM analysis has revealed single-spherical like CNP particles with sizes ranged from 77.9 to 138.1 nm (Figure 6A) while pCNP conferred a size from 68.7 to 144.5 nm (Figure 6B). Similarly, Figure 6C,D indicated an expansion in particle size following PCA encapsulation, where CNP-PCA expanded to a size range of 88.6 to 152.5 nm and pCNP-PCA from 110.6 and 184.1 nm. It was observed that a portion of CNP and pCNP was larger than 100.0 nm, which did not correlate to the DLS results. This was likely to be attributed towards an agglomeration of the nanoparticles. Such a phenomenon has been described as a consequence of the mechanical forces during synthesis. Dogan et al. reported that the aggregation of nanoparticles can occur during drying of samples on the TEM grid prior to observation [73]. Additionally, there exist some nanoparticles with sizes similar to CNP and pCNP observed in Figure 5C,D. These were probably due to the uneven distribution of PCA within CNP and pCNP, where not every CNP and pCNP nanoparticle was encapsulated with PCA resulting in samples that were comprised of empty nanoparticles and encapsulated nanoparticles. Interestingly, the TEM analysis of pCNP showed an additional layer contrast surrounding the inner surface of the outermost layer of pCNP which could be attributed to the palmitoyl groups (fatty acid chains) that was conjugated to the chitosan. This inference was made since this layer was not observed in the TEM analysis of unmodified CNP and it was because the only difference in structure between CNP and pCNP lies is the use of palmitic acid, which suggested this layer was contributed by the conjugation of palmitic acid. Comparing the results obtained between FESEM and TEM, both morphological analyses shown that the nanoparticles appeared single-spherical like in shape. Meanwhile, after encapsulation, the size of the nanoparticles expanded to become larger.

### 3.5. Functional Group Annotation of Nanoparticle Samples Using FTIR Spectroscopy

**Figure 7:**
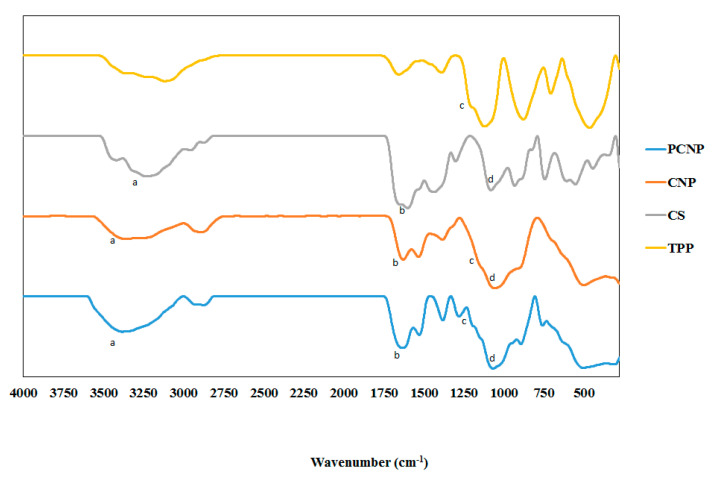
FTIR spectra of CS, TPP, CNP and PCNP have shown some of the important functional group in comparison. The functional groups are labeled as (a) amine group, (b) amine II group, (c) inorganic phosphate group and (d) C–O–C bond.

**Figure 8:**
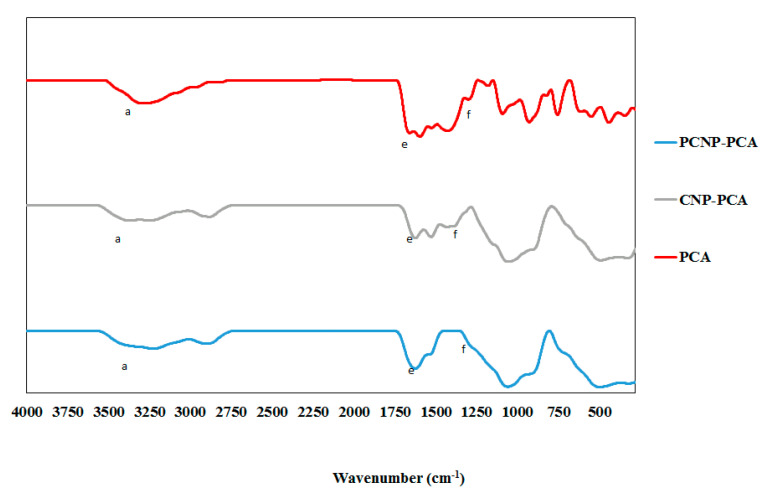
FTIR spectra of PCNP-PCA, PCNP, CNP-PCA, CNP and PCA have shown some of the important functional group in comparison. The functional groups are labeled as (a) hydrogen bond, (e) carbon double bond and (f) carbonyl group.

FTIR analysis was used to annotate chemical functional groups and their occurrences in the nanoparticle samples. According to Coates et al., every single molecule has a unique infrared vibration spectrum which could be their specific “fingerprint” to be identified in a sample by comparing an “unknown” spectrum with the known spectra that had been recorded formerly [74]. The infrared spectra of CS, TPP, CNP and pCNP were listed in Figure 7 while the spectra of CNP, pCNP, CNP-PCA and pCNP-PCA were shown in Figure 8. The important functional groups corresponding to transmittance values of the samples were summarized in Table 2.

In Figure 7, a wide region around 3300 to 3500 nm^−1^ was detected in CS, CNP and PCNP at peaks of 3228, 3360 and 3383 cm^−1^ respectively, which corresponded to hydrogen-bonded O–H stretching and overlapped with primary amine stretching peaks [75]. The IR transmittance of amine group for CS was 49.26% and this increased to 71.24% and 55.82% upon CNP and pCNP formation, respectively. When the percentage of transmittance (% T) is high, the availability of active functional group is considered to be lower in the sample because fewer active function group is present to absorb the IR spectrum. This suggested that upon formation of nanoparticles, free amine groups in the CS were reduced leading a higher transmittance value in CNP and pCNP. This observation also corresponded well to TNBS assay data, showing a utilization of amine groups in chitosan. Additionally, the utilization of amine groups was also suggested by the differences shown by the characteristic peak of amine II group in the range of spectra between 1590 to 1650 cm^−1^. It was observed that about 10% of transmittance at 1600 nm^−1^ for CS increased to 45.68% transmittance at 1629 cm^−1^ and 35.77% T at 1635 cm^−1^ for CNP and pCNP, respectively. The characteristic peak for phosphate groups (P=O) of TPP (1201 cm^−1^) at 35.74% transmittance increased to 40.22% transmittance at 1155 cm^−1^ in CNP and 74.94% transmittance at 1281 cm^−1^ in pCNP; a similar observation was recorded previously by Martin et al. [76]. Next, the vibration of C–O–C stretching was found in CS at 1082 cm^−1^ with 32.20% transmittance, CNP at 1059 cm^−1^, and pCNP at 1068 cm^−1^, with 10% transmittance values [77].

Conversely, PCA exhibited numerous band peaks including functional groups at 3278 cm^−1^ (62.69% transmittance), 1658 cm^−1^ (15.56% transmittance), and 1300 cm^−1^ (69.14% transmittance), which was annotated for hydrogen bonding (O–H) stretching vibrations, C=C stretching, and carboxyl groups (C=O), respectively [78,79]. After PCA encapsulation, the transmittance of the O–H bond peaks increased to 75.50 and 71.13%, occurring at 3376 and 3227 cm^−1^ for CNP-PCA and pCNP-PCA, respectively. Transmittance of C=C bond peaks was also increased to 47.81% at 1625 cm^−1^ for CNP-PCA and 39.61% at 1625 cm^−1^ for pCNP-PCA. The C=O bond was found at 1389 cm^−1^ for 66.75% transmittance for CNP-PCA and at 1280 cm^−1^ for 73.83% transmittance for pCNP-PCA due to the presence of PCA. These results were comparable with the study of Usman et al. where the presence of several functional groups of PCA were found in the nanoparticles after encapsulation [80].

### 3.6. Assessment of In Vitro Cytotoxicity of CNP and pCNP in A549 Lung Cancer Cells

**Figure 9:**
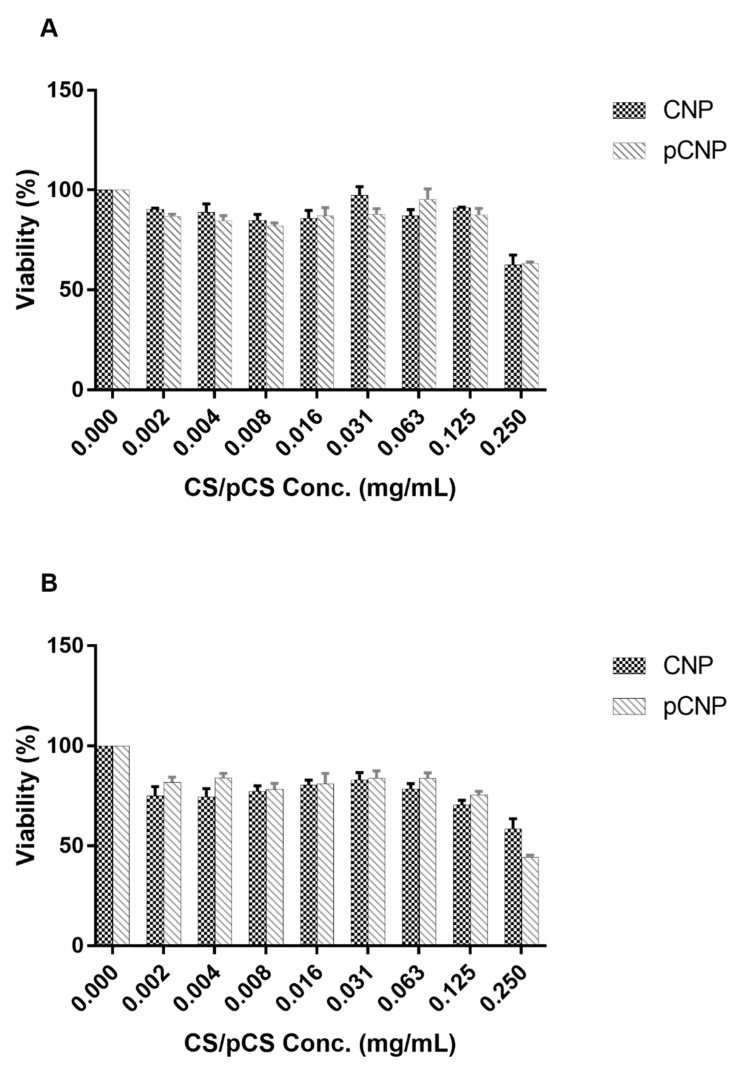
The cytotoxicity effect of CNP and pCNP at (**A**) 24 h post-treatment and (**B**) 72 h post-treatment. Data are presented as mean ± SEM from three independent experiment replicates. Two-tailed paired *t*-test was performed and *p* > 0.05 was obtained indicating no significant different between CNP and pCNP.

CNP has been reported as a good biocompatible nanocarrier system [81,82]. In order to evaluate whether its hydrophobically-modified complement, pCNP is biocompatible as well, MTT cytotoxicity assay was performed against the A549 lung cancer cells. Figure 9 presents the cytotoxicity effects of CNP and pCNP in 24-h and 72-h treatments. The viability of A549 cells 24-h post-treatment after exposure to CNP and pCNP showed similar cytotoxic efficacies, which were 62.62% and 63.25% at the highest nanoparticle concentration of 0.25 mg/mL. At lower concentrations, the viability of cells was at least 80%. A similar cytotoxic effect was attained in 72-h treatments with a slightly lower viability recorded using pCNP compared to CNP at the highest concentration, which was 58.29% and 44.01%, respectively. This suggested that both nanoparticle systems may possess minimal cytotoxicity to the A549 cells. As CNP has been reported to be non-toxic to cells, cytotoxicity was possibly due to the number of nanoparticles that were formed by this parameter [83]. In higher concentrations of CNP/pCNP, the number of nanoparticles synthesized will be greater. This leads to cells being physically covered by the nanoparticles and subsequently cell death. The presence of high nanoparticle populations will easily agglomerate at the cell surface and consequently influence the absorption of nutrients and gaseous exchange and thus confer toxicity to cells [84] Additionally, previous studies have shown that CNP has no any significant cytotoxicity toward several cell lines such as HepG2 human liver cancer cell line, RAW 264.7 mouse macrophage, as well as the A549 cell lines [85,86,87].

The cytotoxicity of nanoparticles is always one of the major aspects to ascertain before being utilized as nanocarrier, especially for nanomedicine applications. Several aspects were taken into consideration when nanoparticles were employed as nanomedicine, such as the cell type of target, the properties of nanoparticle, and the dosage [87]. There are variations in cell physiology, proliferation state, membrane characteristics and phagocyte characteristics exist between different cell types which will have different reaction towards the nanomaterials [88]. Besides that, different sizes and shapes of nanoparticles demonstrated various biokinetic and biological impacts which consequently alter protein adsorption, cellular uptake, accumulation in organelles, and distribution of the body [89,90]. Hence, the optimization of the nanoparticle systems should be performed from time to time when encountering different cell lines.

### 3.7. Assessment Of PCA Efficacy and Anticancer Properties Using Nanoparticle-Mediated In Vitro Cellular Delivery Systems

**Figure 10:**
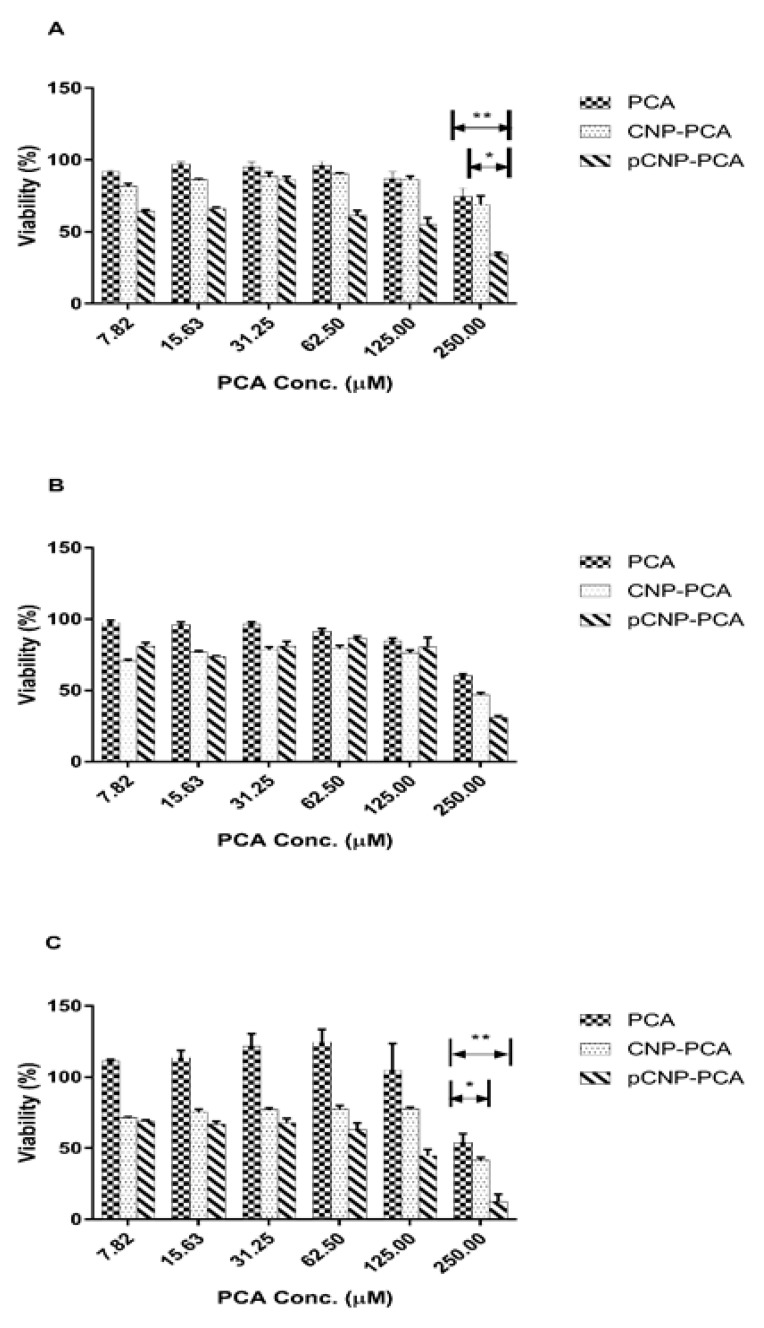
Cellular efficacy of PCA, CNP-PCA and pCNP-PCA at (**A**) 24 h post-treatment, (**B**) 48 h post-treatment and (**C**) 72 h post-treatment on A549 cell line. Data are presented as mean ± SEM from three independent experiment replicates. One-way ANOVA was performed and the significance was indicated by * = *p* < 0.05 and ** = *p* < 0.01 for all three graphs.

The cytotoxic efficacy of PCA, CNP-PCA and pCNP-PCA at different periods of time was tabulated in Figure 10. Approximately 500 µM of PCA was utilized for the encapsulation into CNP and pCNP, and halved after mixing with the media. As shown in Figure 10, the cytotoxic efficacy of the free PCA, CNP-PCA, and pCNP-PCA was shown according to the viability of A549 cell line against the concentration of PCA calculated according to the encapsulation efficiency (35.20 ± 1.71% and 54.39 ± 3.96% for CNP-PCA and pCNP-PCA respectively). In order to compare the efficacy among the three treatments, the IC_50_ values were calculated and are shown in Table 3. The results have shown that CNP-PCA and pCNP-PCA have significantly greater efficacy as compared with the non-encapsulated counterpart. Besides that, pCNP-PCA has achieved the lowest IC_50_ values in 24-h time point and 72-h time point of treatment but slightly lower than CNP-PCA in 48-h time point. At highest PCA concentration, the lowest % cell viability was revealed by pCNP-PCA with 34.25 ± 1.04% viability, followed by CNP-PCA (74.38 ± 1.05%) and free PCA (69.21 ± 1.70%) at 24-h time point. The % viability was subsequently dropped to 30.90 ± 2.37%, 46.76 ± 1.46% and 60.22 ± 1.43% for pCNP-PCA, CNP-PCA and PCA, respectively at 48-h time point. The % viability has further dropped to 12.54 ± 0.88%, 41.54 ± 0.65% and 53.83 ± 1.21% for pCNP-PCA, CNP-PCA, and PCA, respectively, at the 72-h time point. It was observed that PCA delivered by pCNP has the lowest % viability at all three time points and consistently dropped with increased time points. On the other hand, the IC_50_ values tabulated in Table 3 have showed that at 24-h time point, no IC_50_ value was detected for PCA alone and CNP-PCA, while pCNP-PCA was found at around 214.5 μM. Next, there is no IC_50_ found at 48 h post-treatment, as well for PCA alone, but IC_50_ of 448.4 and 412.1 μM were found for CNP-PCA and pCNP-PCA, respectively. After that, at 72 h post-treatment, IC_50_ was calculated at 407.3 and 130.7 μM for CNP-PCA and pCNP-PCA, and no IC_50_ was found for PCA alone.

From the results above, we can deduce that the efficacy of nanoparticle-encapsulated PCA has an undeniably greater cytotoxic efficacy than non-encapsulated counterpart. These consequences may be due to the greater encapsulation efficiency of pCNP than CNP where a greater amount of PCA was encapsulated in pCNP than CNP. Moreover, the hydrophobic-hydrophobic interaction between pCNP and PCA maybe another factor that contributed to the slower release of PCA from pCNP than the conventional CNP that has no specific interaction with PCA [91]. The PCA encapsulation by chitosan nanoparticles has been previously characterized by Madureira and colleagues, and it was found that the bioavailability of PCA was enhanced by CNP encapsulation [92]. Besides that, Pham and coworkers have discovered that the PCA encapsulation by CNP has a greater effect in antifungal activity as compared with the non-encapsulated counterpart which further ascertained that encapsulation of PCA by nanoparticles can greatly enhanced its therapeutic efficacy [93]. The previous study conducted by Barahuie et al. performed nano-encapsulation of PCA by using zinc/aluminium-layered double hydroxide and assessed the cytotoxicity effect on human cervical, liver and colorectal cancer cell lines, and revealed that the anticancer efficacy of nano-encapsulated PCA was greater than the non-encapsulated PCA [79]. Fascinatingly, the IC_50_ of pCNP has no obvious orderly decreasing trend which could attributed by its controlled-release properties. The previous study of Hassan et al. has proposed that fluorescently labeled glutamic acids encapsulated CNP has revealed intracellular release and controlled accumulation properties that was coincided with our current study [94]. Anyhow, the study proposed that the in vitro cellular efficacy of the experimental samples on A549 cell line can be defined in this particular manner: PCA < CNP-PCA < pCNP-PCA.

## 4. Conclusions

The utilization of the nanoparticulate delivery of poorly water soluble, naturally occurring phenolic compounds by using the hydrophobically modified chitosan nanoparticle system has revealed a greater cytotoxic efficacy towards the A549 human lung cancer cell line. In this current research, we found that the conjugation of NHS-palmitic acid to the chitosan has developed into a promising tool for the encapsulation of low water soluble phenolic compounds and PCA, which has a similar cytotoxic effect with the traditional chitosan counterpart but greater encapsulation efficiency and cytotoxic efficacy. Henceforth, this hydrophobic modification system perhaps presents a potential prominent delivery vector that could be customized for the delivery of low bioavailability cancer therapeutics.

## Figures and Tables

**Table 1:** Encapsulation efficiency (*% EE*) of 500 µM PCA in CNP-PCA and pCNP-PCA nanoparticles. Efficiency of the phenolic compound in pCNP-PCA was higher than CNP-PCA due to its active hydrophobic-hydrophobic interaction with palmitoyl in the hydrophobically-modified nanoparticle. Data are presented as mean ± SEM from three independent experiment replicates.

Sample	Free PCA	CNP-PCA		pCNP-PCA	
	*A*_296nm_	*A*_296nm_	*% EE*	*A*_296nm_	*% EE*
Replicate 1	0.79	0.53	32.91	0.42	46.84
Replicate 2	0.83	0.51	38.55	0.33	60.24
Replicate 3	0.82	0.54	34.15	0.36	56.10
Average			35.20 ± 1.71		54.39 ± 3.96

**Table 2:** The chemical functional groups present in CS, TPP, PCA, CNP, PCNP, CNP-PCA and PCNP-PCA. All the important functional groups detected were listed in the table with their respective wavenumber and percentage of transmittance.

Functional Group	Wavenumber (nm^−1^)	Percentage Transmittance (% T)	Sample
Hydrogen bond ^a^ (O—H)	3228	49.26	CS
	3360	71.24	CNP
	3383	55.82	PCNP
	3278	62.69	PCA
	3376	75.50	CNP-PCA
	3227	71.13	PCNP-PCA
Amine II group ^b^ (NH_2_)	1600	10.00	CS
	1629	45.68	CNP
	1635	35.77	PCNP
Inorganic Phosphate ^c^ (P=O)	1201	35.74	TPP
	1155	40.22	CNP
	1281	74.94	pCNP
Ether group ^d^ (C-O-C)	1082	32.20	CS
	1059	10	CNP
	1068	10	pCNP
Carbon double bond ^e^ (C=C)	1658	15.56	PCA
	1625	47.81	CNP-PCA
	1625	39.61	pCNP-PCA
Carbonyl group ^f^ (C=O)	1300	69.14	PCA
	1389	66.75	CNP-PCA
	1280	73.83	pCNP-PCA

Annotations a–f reflects the assigned peaks as indicated in Figure 7 and Figure 8.

**Table 3:** The IC_50_ values of different PCA treatments on A549 cell lines. The encapsulated PCA has greater efficacy than free PCA throughout all time points. Lower IC_50_ values indicate a higher cytotoxic efficiency.

Time Point	24 h	48 h	72 h
	* IC_50_ value (µM)		

PCA	N/A	N/A	N/A
CNP-PCA	191.50	75.78	63.27
pCNP-PCA	53.71	110.70	48.34

* the values were obtained through best-fit hypothetical calculation.
